# Let it bloom: cross‐talk between light and flowering signaling in Arabidopsis

**DOI:** 10.1111/ppl.13073

**Published:** 2020-02-27

**Authors:** Giorgio Perrella, Elisa Vellutini, Anna Zioutopoulou, Eirini Patitaki, Lauren R. Headland, Eirini Kaiserli

**Affiliations:** ^1^ Institute of Molecular, Cell and Systems Biology, College of Medical, Veterinary and Life Sciences University of Glasgow Glasgow G12 8QQ UK; ^2^ ENEA – Trisaia Research Centre 75026 Matera Italy

## Abstract

The terrestrial environment is complex, with many parameters fluctuating on daily and seasonal basis. Plants, in particular, have developed complex sensory and signaling networks to extract and integrate information about their surroundings in order to maximize their fitness and mitigate some of the detrimental effects of their sessile lifestyles. Light and temperature each provide crucial insights on the surrounding environment and, in combination, allow plants to appropriately develop, grow and adapt. Cross‐talk between light and temperature signaling cascades allows plants to time key developmental decisions to ensure they are ‘in sync’ with their environment. In this review, we discuss the major players that regulate light and temperature signaling, and the cross‐talk between them, in reference to a crucial developmental decision faced by plants: to bloom or not to bloom?

**Abbreviations**
AP1: APETALA 1
ARP6: ACTIN‐RELATED PROTEIN 6
bHLH: basic helix–loop–helix
BR: brassinosteroid
bZIP: basic leucine zipper domain
BZR1: BRASSINAZOLE‐RESISTANT1
CDFS: CYCLING DOF FACTORS
CO: CONSTANS
COL4: CONSTANS‐LIKE‐4
COP1: CONSTITUTIVE PHOTOMORPHOGENIC 1
Cry: cryptochrome
EC: Evening Complex
ELF3: EARLY FLOWERING 3
ELF6: EARLY FLOWERING 6
FKF1: FLAVIN‐BINDING KELCH REPEAT F BOX 1
FLC: FLOWERING LOCUS C
FLM: FLOWERING LOCUS M
FRI: FRIGIDA
FT: FLOWERING LOCUS T
FUL: FRUITFUL
GA: gibberellic acid
H3K27me3: tri‐methylation at the 27th lysine residue of histone H3
H3K9: histone 3 lysine 9
HY5: ELONGATED HYPOCOTYL 5
HYH: HY5 HOMOLOG
ICE1: INDUCER OF CBF EXPRESSION 1
JMJ13: JUMONJI 13
LD: long days
LKP2: LOV KELCH PROTEIN 2
miR156: microRNA‐156
PFP: PHD FINGER DOMAIN CONTAINING PROTEIN
phy: phytochrome
PIFs: PHYTOCHROME INTERACTING FACTORS
PRC2: POLYCOMB REPRESSIVE COMPLEX 2
R/FR: red/far‐red light
REF6: RELATIVE OF EARLY FLOWERING 6
RUP: REPRESSOR OF UV‐B PHOTOMORPHOGENESIS 1 AND 2
SAM: shoot apical meristem
SD: short days
SOC1: SUPPRESSOR OF OVEREXPRESSION OF CONSTANS 1
SPA1: SUPPRESSOR OF PHYA‐105
SVP: SHORT VEGETATIVE PHASE
TEM1: TEMPRANILLO 1
TFL1: TERMINAL FLOWER 1
TFS1: TARGET OF FLC AND SPV
TSF: TWIN SISTER OF FT
TZP: TANDEM ZINC KNUCKLE PLUS3
UV‐B: ultraviolet‐B
UVR8: UV‐RESISTANCE LOCUS 8
VIL1: VIN3‐LIKE‐1
VIN3: VERNALIZATION INSENSITIVE 3
VOZ: VASCULAR ONE ZINC FINGER
WRKY75: WRKY DNA BINDING PROTEIN 75
ZTL: ZEITLUPE

## Introduction

Plants undergo a number of developmental transitions throughout their lives that are accompanied by wide‐scale changes in morphology, physiology and metabolism. Major developmental transitions include those associated with seedling germination and growth (including photomorphogenesis), the switch from juvenile to adult forms (vegetative phase change) and from vegetative to reproductive growth (flowering). The timing of these developmental transitions is critical, and plants use both endogenous and exogenous cues to coordinate these processes. In particular, light and temperature are two highly variable and physiologically important parameters that shape plant architecture, growth and productivity. Here, we review recent findings linking light and flowering signaling components with a focus on warm and low temperatures. More specifically, the signaling cross‐talk between light perception and vernalization as well as thermomorphogenesis is discussed.

## Regulation of plant development by light

Light is a powerful informational tool allowing plants to build a comprehensive picture of the current (and likely future) environment they inhabit. At the simplest level, light can vary in quantity, i.e. how much photosynthetically active radiation is absorbed, but variations in direction, duration and quality (i.e. the wavelengths perceived) are all potentially informative. As such, these factors and their fluctuations can be used to provide information concerning time of day (quality, quantity and direction of light perceived), season (day length, i.e. photoperiod) and even presence of neighboring plants (changes in quality). All of these signals can be integrated with other external (e.g. temperature) and internal (e.g. circadian clock) cues, allowing plants to respond to environmental changes and ultimately maximize fitness.

To sense current light conditions, and trigger corresponding downstream responses, plants have evolved a range of photoreceptor proteins. In Arabidopsis, five families of photoreceptors have been characterized. The phytochromes (phyA‐E) primarily absorb and trigger responses relating to the red and far‐red portions of light as well as changes in ambient temperature (Legris et al. 2019). Towards the opposite end of the visible spectrum, the cryptochromes (cry1 and 2), phototropins (phot1 and 2) and the Zeitlupe family [(ZEITLUPE (ZTL), FLAVIN‐BINDING KELCH REPEAT F‐BOX 1 (FKF1) and LOV KELCH PROTEIN 2 (LKP2)] are all mainly associated with blue‐light/UV‐A radiation responses (Christie et al. 2015). Lastly, the most recently characterized photoreceptor, UV‐RESISTANCE LOCUS 8 (UVR8), is responsible for UV‐B specific responses, including those associated with low‐level non‐damaging UV‐B (Yin and Ulm 2017). Evidence suggests that green light may also be perceived by plants, with a potential role for the cryptochromes in this process, however the downstream signaling components and phenotypic effects are relatively less known compared to the other portions of the spectrum (Battle and Jones 2020).

In all these photosensory systems, absorption of light can lead to changes in kinase activity, conformation and/or interactions with other proteins. Often this is also accompanied by changes in subcellular localization; with the downstream signaling events ultimately converging in the nucleus, where different light signals are integrated along with other stimuli (Wu 2014). The result of this complex network of interactions involves large‐scale changes in gene expression, including alterations at the levels of splicing, translation and epigenetic modifications (Wu 2014). Some of the earliest developmental changes in plants occur during germination and seedling establishment, with light playing a major role in regulating these events. The initial process of light‐dependent germination is mediated by the phytochrome photoreceptors, and they are able to do this even after very brief or low light exposure (Sullivan and Deng 2003). The bulk of this responsibility is shared between phyA and phyB in Arabidopsis, with the former regulating very low fluence red, far‐red and continuous red‐light events; and the latter low fluence red‐light dependent germination. Interestingly, both phyA and phyB also appear to be essential for blue‐light dependent germination, while the remaining phytochromes have overlapping roles in select light conditions (Sullivan and Deng 2003).

Shortly after germination, seedlings that have been growing in the dark undergo a dramatic developmental transition known as photomorphogenesis (or alternatively de‐etiolation). Dark‐grown (etiolated) plants generally exhibit a characteristic skotomorphogenic phenotype, including elongated hypocotyls, folded cotyledons and tightly curved apical hook. Light, however, triggers key morphological and physiological changes that prepare seedlings for life in the light. Upon far red/red light exposure, phyA/phyB degrade key negative regulators of growth like the bHLH transcription factors PHYTOCHROME INTERACTING FACTORs (PIFs), thereby releasing the inhibition of photomorphogenesis (Pham et al. 2018). In addition, far red/red‐light‐mediated phyA/phyB, and blue‐light‐mediated cry1/cry2 signaling, converge in their suppression of degradation of the positive regulators of de‐etiolation ELONGATED HYPOCOTYL 5 (HY5) and HY5 HOMOLOG (HYH) by the CONSTITUTIVE PHOTOMORPHOGENIC 1/SUPPRESSOR OF PHYA‐105 (COP1/SPA1) complex (Sullivan and Deng 2003). The resulting morphological response essentially reverses skotomorphogenesis: inhibiting further hypocotyl growth, cotyledon expansion and unfurling of the apical hook. After germination and acclimation to life in the light, plants enter a period of vegetative growth, thereby maximizing their resource capture. Inherent in this stage is a second developmental transition: from juvenile to adult form. In Arabidopsis, this transition is fairly subtle, but is marked by characteristic changes in leaf size and shape (e.g. elongated blades, smooth to serrated margins and presence of abaxial trichomes; Guo et al. 2017). Unsurprisingly, light has been shown to influence the timing of this transition with increases in light accelerating the phase change (Guo et al. 2017).

The last major developmental decision for plants is when to commit to reproduction. It is crucial that this process is carefully managed to ensure it coincides with the optimal conditions for pollination, seed filling and prospects for the future generation. While this area of development has received considerable interest, the complexities of the governing system as well as interactions between other key environmental cues, such as temperature, are continuing to be elucidated.

## Flowering initiation in Arabidopsis

Flowering is one of the most complex regulated pathways in Arabidopsis. To ensure species survival, plants are able to regulate flowering in tune with environmental and endogenous stimuli, including light, temperature and hormones. Arabidopsis flowers preferentially during long spring days (facultative long day plant) but will eventually bolt even in short days when flowering pathways other than the photoperiodic come into play.

Under long day (LD) environmental conditions flowering is mainly controlled through the activation of two florigen components localized within the leaf, FLOWERING LOCUS T (FT) and its paralogue TWIN SISTER OF FT (TSF; Yamaguchi et al. 2005). The photoperiodic pathway is controlled mainly by the zinc‐finger transcription factor CONSTANS (CO). CO binds directly to the *FT* promoter and enhances its expression (Samach et al. 2000). However, during short days (SD), flowering is induced by other environmental cues, as *ft‐1* mutants do not show late flowering phenotypes compared to wild‐type, indicating that not all the floral pathways require florigen genes (Reeves et al. 2002). FT and TSF are mobile proteins able to migrate to the shoot apical meristem (SAM) and initiate the conversion from a vegetative to a floral meristem. FT is detected in the phloem 8 h after a transient induction in the leaf. Once FT and TSF are localized in the SAM, they form a complex with the bZIP transcription factor FD to activate floral meristem identity genes that trigger floral transition, including *APETALA 1* (*AP1*), *SUPPRESSOR OF OVEREXPRESSION OF CONSTANS 1* (*SOC1*) and *FRUITFUL* (*FUL*) (Wigge et al. 2005, Endo et al. 2018). Recent studies using an improved bimolecular fluorescence complementation assay (iBiFC) observed that the interaction between FT and FD occurs primarily in the floral primordium tissue that flanks the meristem cells. Interestingly, soon after floral transition the FT‐FD complex disappears due to lower level of *FD* transcripts in the SAM (Abe et al. 2019).

The simultaneous presence of FT and TSF further strengthens the binding to the promoters of flowering inducing genes such as *AP1* and *FUL* (Collani et al. 2019). Conversely, to prevent the possibility of flowering during unfavorable seasons, negative regulators of flowering such as TEMPRANILLO 1 (TEM1) and FLOWERING LOCUS C (FLC) act antagonistically to FT, by controlling parallel signaling pathways (Golembeski and Imaizumi 2015). In particular, TEM1 acts within the leaf to repress *FT*, while FLC works both within the leaf and in the SAM acting on different levels and targets (Searle et al. 2006, Castillejo and Pelaz 2008).

## Photoperiodic regulation of flowering

Light controls flowering initiation through the action of the photoreceptors that perceive an increase in day length and light quality. The transition from vegetative to reproductive plant growth is highly influenced by the length of the day. In plants, LD ensure an increase in *FT* transcript levels leading to flowering initiation, whilst short days do not allow sufficient accumulation of FT, hence a delay in bolting. To that regard, CO association to the *FT* promoter becomes essential as it occurs in a time‐of‐day‐dependent manner (Casal and Questa 2018).

CO regulation is therefore tightly controlled both at the transcriptional and post‐translational levels (Valverde et al. 2004). *CO* mRNA peaks at the end of the day regardless of the length of the day, under the control of components of the circadian clock. Particularly, CYCLING DOF FACTORS (CDFs) repress *CO* expression in the morning. Under LD conditions, the blue light photoreceptor FKF1 associates with GIGANTEA (GI) and releases *CO* from repression by targeting negative regulators of *CO* expression for degradation (Casal and Questa 2018). As a result, CO levels increase and reach a peak in the late afternoon (Song et al. 2012). Recently, additional light signaling components have been shown to regulate *CO* and *FT* expression, such as TANDEM ZINC KNUCKLE PLUS3 (TZP). PhyB interacts with and recruits TZP to nuclear photobodies where it promotes *FT* and *CO* expression therefore initiating flowering time under LD conditions (Kaiserli et al. 2015).

The difference observed in bolting between SD and LD is also due to CO protein levels during the 24 h period. In the morning, CO is degraded in a phyB‐dependent manner while in the afternoon the blue light photoreceptors phyA, FKF1 and cry2 stabilize CO (Song et al. 2012). In the dark CO is degraded due to the COP1/SPA1 complex (Song et al. 2015).

CONSTANS‐LIKE‐4 (COL4), originally identified as a salt‐stress signaling component has recently been shown to act as a floral repressor (Steinbach 2019). CO and COL4 co‐localize in the nucleus and could act antagonistically allowing a finer regulation of flowering. So far, CO has been linked to control of flowering in LD, however it also has a negative role in flowering induction in SD (Luccioni et al. 2019). CO can inhibit flowering hypothetically through the flowering repressor TFL1, ensuring an alternative mechanism that prevents Arabidopsis from flowering in the wrong season.

A recent paper examined *CO* and *FT* expression in a natural habitat, where the R/FR ratio and daily temperature oscillations are more complex than in laboratory and greenhouse growth conditions used in most experiments. Strikingly, an increase in R/FR to 1 and oscillation in temperature and light regime that mimics natural conditions during summer solstice in Seattle led to a pronounced induction in *CO* and *FT* levels in the morning, unlike what had been observed in routine laboratory conditions (Song et al. 2018). These findings uncover novel regulatory aspects of photoperiodic flowering and open up to the possibility to create experimental conditions mimicking the natural environment that are essential for better understanding the flowering induction process.

## Shade‐induced flowering

Deep plant canopy leads to a decrease in R/FR light ratio, which results in a collection of architectural adaptive responses collectively described as the shade avoidance syndrome (Casal 2013). Acceleration of flowering is one of the shade‐induced responses that allows plants to complete their life cycle and reproduce while growing under competitive environments (Casal 2013). Shade induces an accumulation of PIFs that promote hypocotyl and petiole growth. Moreover, an increase in PIF abundance leads to *FT* and *TSF* induction (Galvāo et al. 2019). In particular, PIF7 together with CO play an additive role in the promotion of flowering under shade conditions through the induction of *FT* expression (Zhang et al. 2019). Further analysis revealed that CO contributes to shade‐induced *FT* activation synergistically with the ATP‐dependent chromatin remodeling factor, PICKLE (Jing et al. 2019).

Recent studies monitoring the effect of shade on the acceleration of flowering in 1001 Arabidopsis accessions revealed that natural variation within the *FT* locus is the main causative agent for shade‐induced flowering (Schwartz et al. 2017). Although not surprising, this study provides additional evidence of the importance of FT in shade‐induced flowering initiation as well as the tools to further study how diverse environmental signals converge in regulating *FT* expression at multiple levels.

## Regulation of flowering induction by UV‐B light

UV‐B is an intrinsic part of sunlight reaching the Earth that ranges from 280 to 315 nm. So far only one genetically encoded UV‐B photoreceptor has been discovered, UVR8, that can sense UV‐B and also mediate responses specific to UV‐B in plants (Yin and Ulm 2017). UVR8 perceives UV‐B directly through the utilization of a specific cluster of tryptophan residues leading to the dissociation of its inactive homodimeric form (Yin and Ulm 2017). Concomitantly, the active UVR8 monomers interact with COP1 and through the stabilization of HY5, regulate the expression of genes involved in light signaling (Yin and Ulm 2017).

The effect of UV‐B radiation and the potential involvement of UVR8 in flowering induction have not been thoroughly investigated and there is limited information on this topic. In various plant species including Arabidopsis, as well as *Phaseolus vulgaris*, *Vigna radiate* and maize, a general delay in flowering has been observed when plants are subjected to various UV‐B fluence rates (Mark and Tevini 1997, Rajendiran and Ramanujam 2004, Yan et al. 2012, Hayes et al. 2014, Del‐Castillo‐Alonso et al. 2016, Arongaus et al. 2018, Dotto et al. 2018). Hayes et al. (2014) investigated the flowering time of wild‐type and *uvr8* mutant lines grown under white light supplemented with UV‐B and demonstrated that UV‐B leads to a delay in flowering in wild‐type and an early flowering phenotype in *uvr8*.

A more recent study by Dotto et al. (2018) further investigated the role of UV‐B in flowering initiation by conducting experiments under both long and short day conditions ± UV‐B and showed that UV‐B radiation causes a delay in the flowering time of wild‐type Arabidopsis. Although these findings were in agreement with the earlier study by Hayes et al. (2014) *uvr8* mutants were found to flower at the same time independently of the UV‐B radiation treatment (Dotto et al. 2018). This difference in the flowering phenotype of *uvr8* could potentially be explained by the variations in the UV‐B conditions used in each study. The aforementioned study also investigated the effect of UV‐B on the autonomous and aging flowering pathways and proposed a mechanism where the two flowering repressors *FLC* and *miR156* are upregulated in a UV‐B‐dependent manner and therefore prolonging the juvenile stage of the plant's growth and inhibiting its transition to a reproductive state (Dotto et al. 2018). The above is a result of decreases in the H3K27me3 repressive mark on *miR156* and *FLC* genes and was found to correlate with a downregulation in the gene expression levels of the floral integrators *FT* and *SOC1* (Dotto et al. 2018).

Previous reports have shown that RUP1 and RUP2 are negative regulators of UV‐B‐induced photomorphogenesis (Yin and Ulm 2017). Interestingly, *rup2* and *rup1rup2* mutants have a UVR8‐dependent early flowering phenotype compared to wild‐type under short day conditions supplemented with UV‐B, implying that RUP2 is a repressor of UVR8‐induced flowering (Arongaus et al. 2018). In particular, under SD + UV‐B, RUP2 interacts directly with the flowering‐promoting protein CO and represses its activity, which results in decreased levels of *FT* transcript and delayed flowering (Arongaus et al. 2018). Altogether this work describes how UV‐B could determine flowering in plants. However, how natural UV‐B regimes regulate flowering initiation still remains to be uncovered.

## Regulation of flowering by hormones

In addition to light and temperature, hormones play an important role in the control of flowering initiation. For example, mutations that reduce gibberellic acid (GA) biosynthesis cause a delay in flowering under LD whilst almost abolishing it under SD. The CO antagonist TEM1 inhibits GA production by binding to the Transcriptional Start Site (TSS) of the GA_4_ biosynthetic genes *GA 3–oxidase1* and *2* thereby reducing their expression. Furthermore, transgenic lines overexpressing *TEM1* resemble GA‐deficient mutants (Osnato et al. 2012) placing TEM1 at the cross‐talk between GA and photoperiodic pathways. Meanwhile, the transcription factor WRKY75 promotes flowering in a GA‐dependent manner. In particular, through its interaction with DELLA proteins, WRKY75 contributes to the regulation of flowering by binding to the *FT* promoter when plants are supplemented with GA_3_ (Zhang et al. 2018). DELLA proteins are also direct interactors of PIF proteins. Under shade, an increase in GA leads to a breakdown of DELLAs, thereby allowing PIFs to activate gene transcription (Yang and Li 2017). Brassinosteroids (BR) are steroidal hormones that regulate different stages of plant development. In Arabidopsis, most BR‐biosynthetic and BR‐insensitive mutants display several anomalies including dwarfism, a prolonged vegetative phase and delayed flowering time (Li et al. 2010). In particular, BRs regulate flowering time by modulating *FLC* expression through the autonomous pathway. However, recent studies have shown BRs also acting as negative regulators of flowering. Li et al. (2018) demonstrated that the bHLH transcription factor, BRASSINAZOLE‐RESISTANT1 (BZR1) recruits the histone demethylase EARLY FLOWERING 6 (ELF6) on *FLC* chromatin and removes the H3K27me3 repressive marks, hence inducing *FLC* expression and the consequent floral repression. Altogether, these studies have shown that the role of BR in flowering induction still requires further clarification.

## Temperature‐induced flowering initiation

### Vernalization

Exposure to prolonged cold temperature induces flowering by silencing a key flowering repressor, *FLC*, through a process called vernalization. FLC encodes a MADS‐box transcription factor that represses the expression of the flowering inducers *FT* in the leaf and *SOC1* in the SAM (Michaels and Amasino 1999, Searle et al. 2006). Winter temperatures promote *FLC* repression through several epigenetic mechanisms so the plant can flower when spring arrives and therefore conferring a memory of the past winter (Michaels and Amasino 1999).

In particular, during vernalization, *FLC* expression is repressed through the action of long non‐coding RNAs as well as histone methylation mediated by the Polycomb Repressive Complex 2 (PRC2) (De Lucia et al. 2008). The autonomous pathway operates regardless of day‐length and concurs to repress *FLC* together with the vernalization pathway, mainly through RNA processing and chromatin remodeling.

Altogether, FLC offers a very elegant mechanism for sensing temperature oscillations. Recently, Hepworth et al. (2018) have linked the decrease observed in *FLC* during autumn with the perception of sporadic cold peaks (below 14°C). This perception leads to a temporary silencing of *FLC* through antisense transcripts. During winter, the absence of warm peaks (above 15°C) is necessary for reinforcing *FLC* downregulation through epigenetic silencing (Hepworth et al. 2018). FLC and SOC1 have an antagonistic effect on flowering and their effects on the newly identified bolting promoting gene *TARGET OF FLC AND SPV1 (TFS1)* have been investigated. Specifically, FLC represses *TFS1* through the deposition of chromatin marks such as H3K27me3; conversely SOC1 promotes *TFS1* by recruiting RELATIVE OF EARLY FLOWERING 6 (REF6), a histone demethylase, and the chromatin remodeling enzyme BRAHMA (Richter et al. 2019).

### Cross‐talk between photoperiodic‐ and vernalization‐induced flowering

Cold temperatures (vernalization) and the autonomous pathway concur to silence the main repressor of flowering *FLC*, enabling the plant to flower when more favorable environmental conditions come into play. *FLC* is transcriptionally activated by FRIGIDA (FRI). In the common *Arabidopsis thaliana* accession Columbia (Col‐0), FRI is not active because of a premature stop codon. Consequently, Col‐0 does not require vernalization for flowering induction (Shindo et al. 2005). *FLC* is repressed by antisense mRNAs and by epigenetic modifications through the PRC2 together with the PHD containing protein VERNALIZATION INSENSITIVE 3 (VIN3; Hepworth et al. 2018).

**Figure 1:**
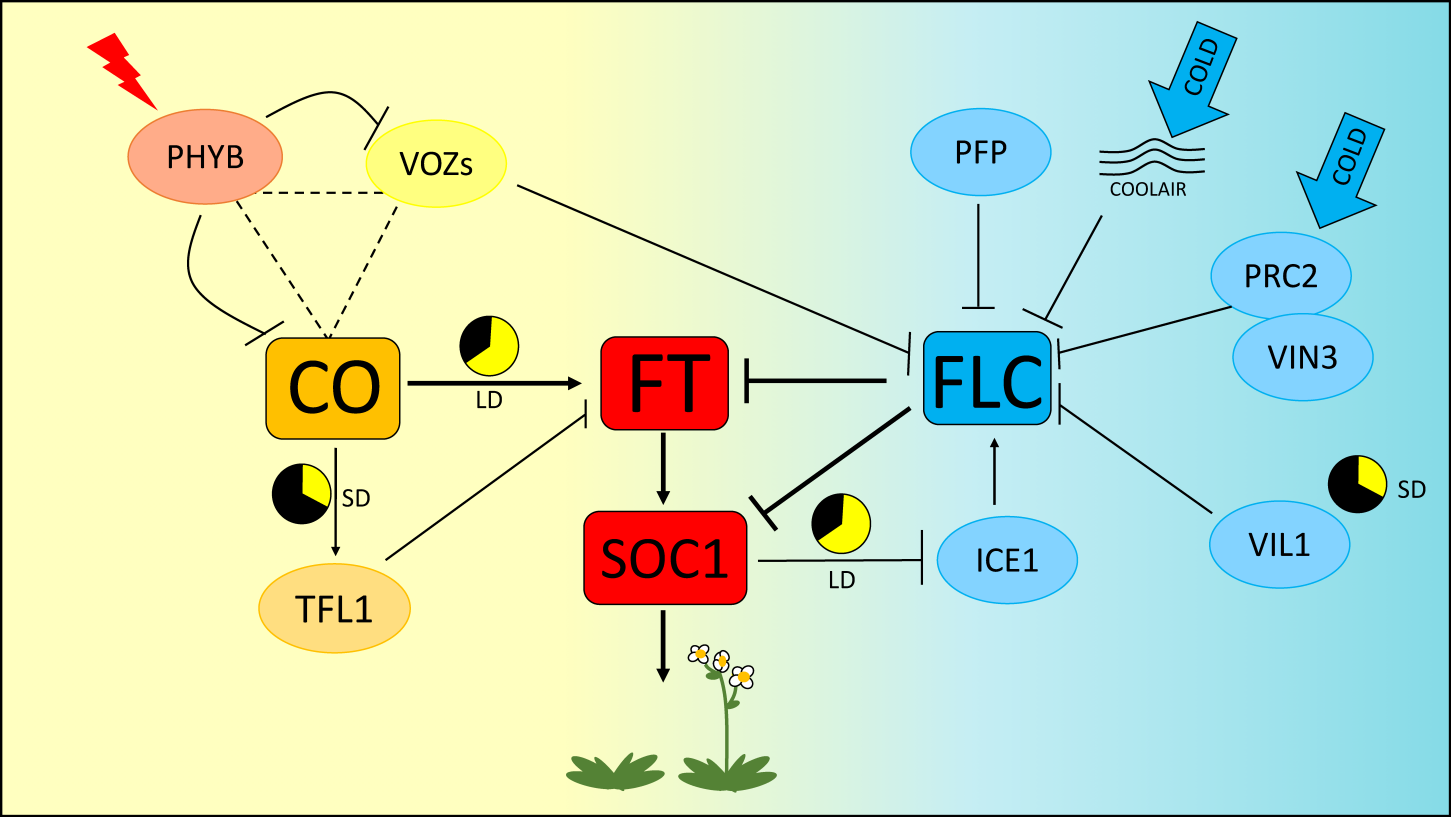
Cross‐talk between vernalization and photoperiodic pathways (model). The main flowering regulators are represented in rectangular boxes, other factors upstream or downstream of the flowering pathways are represented with circles. FLOWERING LOCUS T (FT) acts through the SUPPRESSOR OF OVEREXPRESSION OF CONSTANS 1 (SOC1). *FT* expression is activated under LD by the transcription factor CONSTANS (CO). Under SD, CO acts as a repressor of flowering through the activation of TERMINAL FLOWER 1 (TFL1). Cold temperature silences the FLOWERING repressor *FLOWERING LOCUS C (FLC)* via the antisense mRNA (*COOLAIR*) together with a complex between POLYCOMB REPRESSIVE COMPLEX 2 (PRC2) and VERNALIZATION INSENSITIVE 3 (VIN3). *VIN3‐LIKE 1 (VIL1)* is upregulated during SD and is required to silence *FLC* in response to vernalization. The PHD finger domain containing protein (PFP) concurs to repress *FLC* in a photoperiod‐independent manner. In LD conditions, SOC1 modulates *FLC* expression by repressing the positive regulator *INDUCER OF CBF EXPRESSION 1* (*ICE1*). VASCULAR ONE ZINC FINGER 1 (VOZ1) and 2 (VOZ2) negatively regulate *FLC*. PHYTOCHROME B (PHYB) inhibits VOZs and CO in a time‐of‐day‐dependent manner.

Cross‐talk between temperature and light sensing pathways ensures more efficient adaptation during unpredictable environmental conditions. Early studies have shown that VIN3‐LIKE 1 (VIL1), a PHD containing protein, is involved in both non‐inductive photoperiodic pathway and vernalization. *VIL1* is upregulated during SD and induces flowering through the repression of the negative regulator *FLOWERING LOCUS M (FLM)*. This results in a delay in flowering time during SD in *vil1*. VIL1 also regulates the chromatin state of *FLC*. An enrichment in the repressive marks on the *FLC* locus, primarily H3K9 and H3K27 methylation, occurs in response to vernalization. As a result *vil1* mutant exhibits an increase in *FLC* repressive marks in response to lower temperature resulting in a lack of *FLC* (Sung et al. 2006). More recently, another PHD Finger domain containing Protein, PFP, was newly identified as a repressor of *FLC* but interestingly, in a photoperiod‐independent manner as both loss of function and over expressing lines for *PFP* showed a flowering phenotype in either LD or SD conditions (Fig. 1; Yokoyama et al. 2019).

Recently, two novel phyB‐interacting proteins, VASCULAR ONE ZINC FINGER 1 (VOZ1) and VOZ2 were shown to play a role in both the vernalization and photoperiodic pathways (Yasui and Kohchi 2014, Kumar et al. 2018). *Voz1 voz2* double mutants exhibit a late flowering phenotype and an overexpression of *FLC* in the absence of vernalization. The aforementioned phenotype is suppressed upon exposure to cold temperatures suggesting that VOZ1 and VOZ2 promote flowering through *FLC* repression (Yasui and Kohchi 2014). Conversely, a more recent paper has shown that the *voz1 voz2 flc* triple mutant cannot suppress the late flowering phenotype of *voz1 voz2* suggesting that VOZs act primarily on the photoperiodic pathway, independently of FLC (Kumar et al. 2018). Furthermore, it has recently been shown that VOZs directly interact with CO and promote flowering independently of FLC. A possible explanation for this discrepancy could be due to the FLC‐independent mechanism that promotes flowering through vernalization (Michaels and Amasino 2001, Kumar et al. 2018).

An additional point of cross‐talk between the photoperiodic and vernalization signaling pathways is through the INDUCER OF CBF EXPRESSION 1 (ICE1), which encodes a MYC‐like bHLH transcriptional activator involved in the cold temperature response. ICE1 regulates both *FLC* and *SOC1* expression and therefore integrates cold responses to the photoperiodic pathway. ICE1 binds directly the *FLC* promoter when exposed to cold temperatures, leading to delayed flowering. Under LD photoperiods SOC1 inhibits ICE1 from activating *FLC* (Fig. 1; Lee et al. 2015).

VOZ1, VOZ2 and ICE1 transcription factors offer a novel mechanism that could enlighten how different environmental cues are integrated into flowering time control. However, the exact mechanism and point of integration between cold temperature and photoperiod in modulating flowering requires further investigation.

### Photoperiod and warm temperature mediate flowering

Temperature is an important environmental factor that regulates the transition from a vegetative to a reproductive growth in Arabidopsis. Over the next few decades, temperature is set to rise due to global warming, clearly affecting the flowering process.

**Figure 2:**
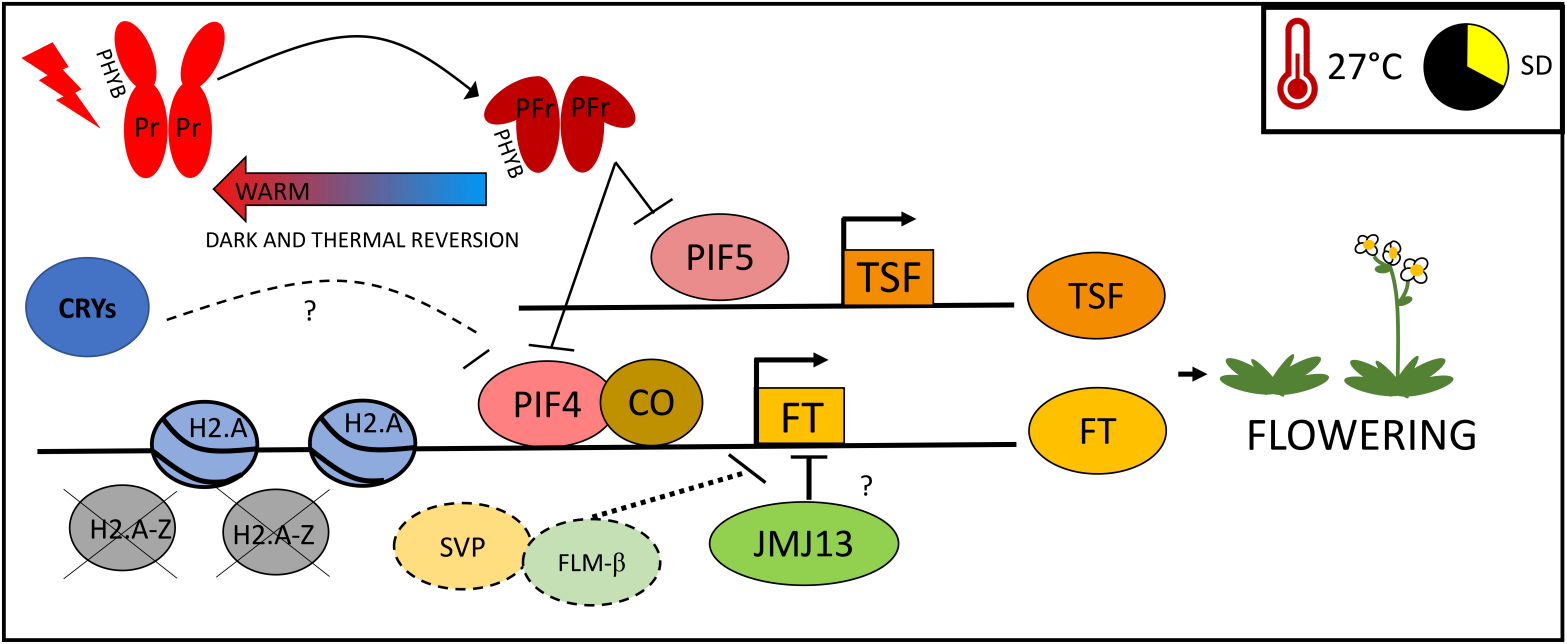
Warm temperatures induce flowering initiation (proposed model). Genes are represented in rectangular boxes, proteins are represented in circles. Non‐inductive photoperiodic conditions (SD) coupled with warm temperatures (27°C) induce flowering in Arabidopsis. PHYTOCHROME INTERACTING FACTOR 4 (PIF4) AND 5 (PIF5) are repressed by CRYPTOCHROMES (CRYs) and by the active far‐red absorbing (Pfr) form of PHYTOCHROME B (PHYB). At 27°C the Pfr form is rapidly converted to the inactive Pr form thus releasing PIFs from PHYB‐dependent repression. Under warm temperatures, CO physically interacts with PIF4 and bind to the *FLOWERING LOCUS T* (*FT*) promoter. Similarly, PIF5 is located on *TWIN SISTER OF FT* (*TSF*) promoter, thereby promoting flowering induction. Moreover, at 27°C, the repressive histone variants H2A.Z are exchanged with the permissive H2A containing nucleosomes hence allowing PIF4 to bind effectively to the *FT* promoter. Warmer temperatures induce the alternative splicing variant of *FLM*, FLM‐β, which destabilizes the complex SHORT VEGETATIVE PHASE (SVP) and FLOWERING LOCUS M (FLM) that negatively regulate *FT*. The H3K27 demethylase JUMONJI 13 (JMJ13) represses flowering during warm SD due to a reduction in *FT* repression.

Unlike lower temperatures that are mainly linked to a delay in flowering, warmer temperatures (27°C) promote the expression of the florigen *FT*, thereby leading to the reduction of flowering time (Kumar et al. 2012). Early studies have shown that flowering initiation at warm temperature is mainly mediated by the bHLH transcription factor PIF4 (Kumar et al. 2012). Arabidopsis plants flower preferentially during LD photoperiods, however, exposure to warm temperature during short day conditions is sufficient to restore early flowering time. PIF4 is required for flowering at warm temperature under SD: at 27°C *pif4* mutants do not show acceleration in flowering observed in the WT. In addition, chromatin immunoprecipitation experiments showed that PIF4 can directly bind the *FT* promoter demonstrating that PIF4 is required for *FT* induction in a temperature‐dependent manner (Fig. 2; Kumar et al. 2012). The induction of *FT* at warm temperature also requires a permissive chromatin environment. Consistent with this hypothesis, Kumar et al. (2018) found that the levels of H2A.*Z*‐nucleosomes at the *FT* promoter decrease in response to warmer temperature. Similarly, PIF4 binding to *FT* promoter was greater in the absence of ACTIN‐RELATED PROTEIN (ARP6), which is responsible for nucleosome exchange between H2A.Z and H2A (Kumar and Wigge 2010).

Recently it was showed that phytochromes associate with the promoter of temperature‐responsive genes, in line with the fact that phyB functions as a thermosensor (Jung et al. 2016). However, unlike PIF4, the binding of phyB to promoter regions of genes that are primarily expressed during the night was decreased at warm temperature (Jung et al. 2016). Recent studies have shown that members of the Evening Complex (EC) such as EARLY FLOWERING 3 (ELF3) and phytochromes present additive functions, as quadruple mutants between *elf3‐1* and *phyBDE* showed elongated hypocotyls compared to the respective mutants, making it therefore likely that temperature information can be transmitted through the EC also during flowering initiation (Ezer et al. 2017). Similar to PIF4, PIF5 is able to promote flowering at warm temperatures in SD acting through the *FT* paralogue *TSF* (Fernández et al. 2016). Histone erasers such as the histone demethylase JUMONJI 13 (JMJ13) can also repress flowering primarily at 27°C under SD conditions through a cross‐talk between JMJ13, CO and GI (Fig. 2; Zheng et al. 2019).

Moderate increases in temperature can also activate flowering time in Arabidopsis through the interaction between MADS‐domain proteins FLOWERING LOCUS M (FLM) and SHORT VEGETATIVE PHASE (SVP). This complex is able to repress flowering at low ambient temperatures, however, an increase in temperature destabilizes SVP protein and induces the formation of a non‐functional splicing variant of *FLM*, thereby affecting the stability and abundance of the SVP–FLM complex (Fig. 2; Verhage et al. 2014).

Elevated temperatures can also trigger the nuclear translocation of COP1, where it targets HY5 for degradation, hence allowing hypocotyl elongation at warm temperatures (Park et al. 2017). The *cop1‐4* mutant was previously shown to display a photoperiod‐insensitive flowering phenotype due to altered circadian rhythms (Yu et al. 2008). It would be interesting to investigate how temperature may influence COP‐regulated flowering initiation. Additionally, recent work has shown thermo‐sensory responses for photoreceptors and light signaling components regulating hypocotyl growth (Ma et al. 2016, Huang et al. 2019). Based on these observations, it becomes now intriguing to assess whether these thermomorphogenesis mechanisms are also conserved during flowering transitions.

Taken together, the integration between light, photoperiod and temperature still needs to be fully uncovered to provide a complete mechanism of how Arabidopsis plants sense and adapt to the environment in order to flower at the right time. Temperature sensing becomes therefore fundamental during unfavorable climate conditions to modulate photoperiodic flowering. As shown previously, PIF4 ensures flowering in warm days. Conversely, JMJ13 could act in an opposite manner by negatively regulating flowering time in a temperature and photoperiod dependent manner.

## Conclusions and future perspectives

One of the keys to success for plant robustness is the careful timing of developmental transitions; up to and including one of the final key decisions prior to senescence – when to flower. Committing to reproductive growth is irreversible and the environment in which flowers are produced, pollinated and set seed, can have far reaching impacts on the next generation. Integration of light and temperature cues allows better plant adaptation and survival.

Our knowledge of plant responses to light and temperature have expanded enormously in the last few decades. However, the molecular mechanism underlying how the photoperiodic, vernalization and thermomorphogenesis signaling pathways converge with regards to regulating flowering initiation still remains elusive. Certain key light signaling components have recently been associated with mediating plant architectural changes in response to elevated ambient temperature; however, their role in the thermo‐regulation of flowering is less clear or not investigated to date.

How prolonged cold temperatures (vernalization) and changes in light quality, fluence rate and duration are integrated at the molecular level has only recently been investigated and lead to the identification of novel signaling components bridging the two pathways. Furthermore, to what extent endogenous factors such as the circadian clock and hormone biosynthesis contribute to light‐ and temperature‐dependent flowering is still not known.

Understanding how signal integration and cross‐talk occurs at the level of perception and signaling is fundamental for elucidating the mechanism of plant adaptation in response to multiple stimuli. Are shared signaling components exclusive to specific developmental stages? Are there additional unidentified proteins that act as bridges between light‐ and temperature‐controlled flowering? Answering such questions is essential prior to applying our knowledge to optimizing plant productivity in response to global climate change.

## Author contributions

G.P., E.V., A.Z., E.P., L.R.H. and E.K. wrote the manuscript. G.P. and E.V. designed and produced the figures. G.P. and E.V. contributed equally to this work. E.K. would like to thank the Biotechnology and Biological Sciences Research Council for the New Investigator grant award BB/M023079/1. E.V. and A.Z. are funded by PhD studentships awarded by the College of Medical, Veterinary & Life Sciences at the University of Glasgow.

## Data Availability

Data sharing is not applicable to this article as no new data were created or analyzed in this study.
